# CircADARB1 serves as a new biomarker in natural killer T-cell lymphoma and a potential regulator of p-Stat3

**DOI:** 10.1186/s12935-021-02296-x

**Published:** 2021-11-04

**Authors:** Mei Mei, Yingjun Wang, Wenting Song, Zhaoming Li, Qilong Wang, Jiayin Li, Mingzhi Zhang

**Affiliations:** 1 Department of Oncology, The First Affiliated Hospital of Zhengzhou University, Νo. 1 Jianshe East Road, Zhengzhou, Henan China; 2 The Academy of Medical Sciences, Zhengzhou University, Zhengzhou, China; 3 Diagnosis and Treatment Center of Lymphoma of Henan Province, Zhengzhou, Henan China

**Keywords:** Noncoding RNAs, Circular RNAs, microRNAs, Natural killer/T-cell lymphoma (NKTCL)

## Abstract

**Background:**

Natural killer/T-cell lymphoma (NKTCL) is a rare and aggressive subtype of Non-Hodgkin’s Lymphoma. CircRNA has shown great potential to become a biomarker in plasma. In this study, we aimed to determine circRNA for its diagnostic and prognostic value and biological function in NKTCL.

**Method:**

The circRNA microarray of plasma from NKTCL patients and healthy donors were conducted. The relative expressions of target circRNA were verified by qRT-PCR. We conducted function experiments in vitro and in vivo. Bioinformatics predicted the target miRNA of the target circRNA and the binding site was detected by the dual luciferase report assay. Downstream target protein was predicted and detected by western blot in vitro and immunohistochemistry in vivo.

**Result:**

By analyzing the plasma circRNA microarrays in NKTCL, 6137 circRNAs were up-regulated and 6190 circRNAs were down-regulated. The relative expressions of circADARB1 were significantly higher in NKTCL patients. The knockdown of circADARB1 inhibited proliferation of NKTCL cells in vitro and in vivo. CircADARB1 could bind to miR-214-3p in the downstream and regulate the expression of p-Stat3. In nude mice tumor tissue, p-Stat3 was under-expressed in the circADARB1 knockdown group.

**Conclusion:**

CircADARB1 was highly expressed in NKTCL plasma and circADARB1 was a potential biomarker to assist diagnosis and predict the response in NKTCL. CircADARB1 bound up to miR-214-3p and regulated p-Stat3.

**Supplementary Information:**

The online version contains supplementary material available at 10.1186/s12935-021-02296-x.

## Introduction

85–90% of Non-Hodgkin’s Lymphoma (NHL) originates from B lymphocytes, the remaining non-Hodgkin Lymphoma originates from T lymphocytes and Natural Killer (NK) lymphocytes [1]. Natural killer/T-cell lymphoma (NKTCL) is a rare and aggressive subtype of NHL originating from NK lymphocyte and cytotoxic T lymphocyte [2] and associated with Epstein-Barr virus infection [3]. NKTCL is endemic in East Asia and Latin America while rare in Europe and South America [4], usually associated with a poor outcome [5]. Due to its atypical early clinical manifestations, including general symptoms such as fever, night sweats, and fatigue, as well as related symptoms involving organs. The current diagnosis mainly depends on immunohistochemical staining, and the immunohistochemical characteristics of NKTCL cells include CD2 and CD56 positive, CD3 negative; cytoplasmic CD3ε and cytotoxic molecules (perforin, granzyme, TIA1) positive [6]. The prognostic index of natural killer cell lymphoma (PINK) and PINK-E [7] includes age greater than 60 years, stage 3 or 4 stage, involving distant lymph nodes and non-nasal diseases, and the addition of EBV DNA testing .

However, it is difficult to distinguish NKTCL since the early symptoms are not typical. Tumor tissue necrosis is common, which makes sampling difficult and misdiagnosed. In China, patients with pathological test of NKTCL more than once account for 37.1% [8]. In Western countries, the median time from symptom onset to diagnosis is 5 months, the longest is 36 months, and patients with more than once pathological examination account for 48% of patients with NKTCL [9]. Therefore, it is imperative to find new markers to assist in tumor diagnosis.

In addition, efficacy prediction and therapeutic targeting are extremely important in molecular tumor research. Wang et al. found that about 95% of ENKTL cases were CD38 positive and that patients with strong CD38 expression had a low CR rate after chemotherapy [10]. A subsequent clinical trial showed that daratumumab, a monoclonal antibody against CD38, achieved an ORR of only 25% in patients with R/R NKTCL, and none of the patients achieved CR [11]. Although there have been many molecular studies on NKTCL, there is still a lack of validated efficacy predictive biomarkers and therapeutic targets.

Circular (circ)RNA specializes in forming covalently closed continuous loops with back-splicing events [12]. The majority of circRNAs are generated from coding exons (usually between one and five exons) [13, 14]. CircRNAs were initially considered as RNA by-products [15]. Recent studies have revealed biological functions as miRNA sponges [14], regulating transcription or splicing [16, 17], and interacting with RNA binding proteins (RBPs) [18].

CircRNA has shown great potential to become a biomarker because of its conserved, stable, and stage- and tissues-specific characteristic [19, 20]. In addition, circRNAs exist widely in human body, not only in tissues, but also in the human peripheral blood [21, 22] and saliva [23] where can be collected easily [24]. In previous researches, several circRNAs in B-cell lymphoma [25, 26] and T-cell lymphoma [27] have been emphasized. However, clinical references for circRNAs profile and biological functions in NKTCL are not clear. In this study, we focused on circRNA for its diagnostic and prognostic value, and biological function in NKTCL.

## Materials and methods

### Patients and samples

The plasma samples of the control group involved in this experiment were obtained from healthy donors, and the plasma samples of the experimental group were obtained from patients with pathological diagnosis of NK/T-cell lymphoma. Inclusion criteria for the experimental group: (1) those who attended the First Affiliated Hospital of Zhengzhou University between 2012 and 2019, (2) patients with confirmed NK/T-cell lymphoma diagnosed or consulted by the Department of Pathology of the First Affiliated Hospital of Zhengzhou University, (3) availability of all clinical data, and (4) plasma samples collected at the first admission and signed an informed consent form. Exclusion criteria: (1) patients were previously treated with antineoplastic therapy, (2) patients with an unclear pathological diagnosis or a diagnosis of mixed malignant lymphoma. Clinical data such as age, gender, B symptoms, staging [28], PINK-E, ECOG score, response to therapy and follow-up information were collected.

The initial study involved six patients with a recent diagnosis of NKTCL and six healthy donors as negative controls. Validation experiments involved 50 patients diagnosed with NKTCL and 50 healthy donors as negative controls. Plasma samples from patients were collected before chemotherapy and preserved at − 80 °C until analyzed.

### Total RNA extraction

Total RNA was extracted from plasma samples using TRIzol LS reagent (Invitrogen, Carlsbad, CA, USA) and from cells using TRIzol reagent (Invitrogen) according to the manufacturer’s instructions. The purity and concentration of RNA samples were determined with a Nanodrop 1000 spectrophotometry (Thermo Scientific, Wilmington, DE, USA).

### Circular RNA preparation and microarray

Total RNAs were digested with Rnase R (Epicentre, Inc.) to remove linear RNAs and enrich circular RNAs. Then, the enriched circular RNAs were amplified and transcribed into fluorescent cRNA utilizing a random priming method (Arraystar Super RNA Labeling Kit; Arraystar). The labeled cRNAs were hybridized onto the Arraystar Human circRNA Array V2 (8 × 15 K, Arraystar). After having washed the slides, the arrays were scanned by the Agilent Scanner G2505C.

### Quantitative reverse transcription polymerase chain reaction (qRT-PCR)

cDNA was synthesized by reverse transcription using the PrimeScript RT Master Mix with random primers, and qRT-PCR was performed according to the manufacturer’s protocols (TaKaRa Bio, Shiga, Japan). Levels of circRNA and mRNA were normalized to GAPDH. Relative expression level was calculated with the 2^−ΔΔCt^ method.

### Cell culture

HEK293T and YT cells were cultured in Dulbecco’s Modified Eagle Medium (DMEM) and Roswell Park Memorial Institute (RPMI) 1640, respectively (ThermoFisher, MA, USA) containing 10% fetal bovine serum (Clark Bioscience, VA, USA). SNK-6 cell line was cultured in RPMI 1640 containing 5% advanced cell culture supplement (AventaCell BioMedical Co., GA, USA) and 1000 U/ml recombinant human IL-2. In addition, 100 U/ml penicillin and streptomycin (ThemoFisher Scientific, MA, USA) were added in cell culture medium at 37 °C with 5% CO_2_.

### CircRNA plasmid construction and stable transfection

circADARB1 cDNA was synthesized and cloned into the GV493 vector (Genechem, Shanghai, China) to construct three short hairpin RNA plasmids. The plasmid constructions were confirmed by sequencing, then transfected into HEK293T cells for lentiviral packaging using Lipofectamine 2000 (Invitrogen) according to the manufacturer’s protocol. After the lentivirus harvested, NKTCL cell lines YT and SNK-6 were transfected and selected for seven days with 3 μg/mL puromycin.

### Cell viability assay

YT and SNK-6 cells were cultured in 96-well plates coated with 20 μl of Cell Counting Kit-8 (CCK-8, Dojindo, Tokyo, Japan) per well and incubated at 37 °C supplied with 5% CO_2_ for 2 h. Next, cell viability was presented by the wavelength of OD450 nm with a Multiskan FC microplate reader (Thermo Scientific, Waltham, MA, USA).

### Cell apoptosis analysis

Cell apoptosis assay was performed using the Annexin V-APC Apoptosis Detection Kit (BD Biosciences, USA) following the manufacturer’s instructions. Cells were stained with Annexin V-APC and 7AAD, followed by flow cytometry in a FACS Canto II (BD Biosciences).

### Bioinformatics analysis

Targetscan (http://www.targetscan.org) [29], ENCORI (formerly known as starBase) [30] and miRanda [31] for bioinformatics analysis and prediction.

### Dual luciferase reporter assay

The different fragment sequences were synthesized and then inserted into the psiCHECK2 vector (Hanbio, China). Luciferase activity was assessed using the Luciferase Assay Reagent II (LAR II) (Luciferase Assay Reagent, Progema) according to the manufacturer’s instructions.

### Western blotting

Total protein was extracted from cells using RIPA with Protease and Phosphatase Inhibitor cocktail (CWBIO, China) and EDTA cocktail. The protein concentrations were detected by BCA Protein Assay Kit (CWBIO, China) according to the manufacturer’s instructions. Electrophoresis was performed with sodium dodecyl sulfate–polyacrylamide gel electrophoresis (SDS-PAGE), and protein was transferred onto polyvinylidene fluoride (PVDF) membranes (Amersham Biosciences, Piscataway, NJ, USA). Primary antibodies used were phospho-Stat3 (1:1000), Bax (1:1000), GAPDH (1:2000) (Cell Signaling Technology, Boston, MA, USA) and the secondary antibodies (ProteinTech, Chicago, IL, USA). Detection was carried out with the eECL Western Blot Kit (CWBIO, China). The band images were digitally captured and quantified with a ChemiDoc. XRS + system (Bio-Rad Laboratories, Hercules, CA, USA).

### In vivo tumor xenograft

Ten female BALB/c nude mice (6-week-old) were maintained under specific pathogen-free conditions from the Nanjing Medical Experimental Animal Care Commission. Transfected YT cells were harvested into two groups. 1 × 10^7^ cells were subcutaneously injected into a single flank of each mouse. The mice were CO_2_ euthanasia at a rate of 30% chamber volume/min using an euthanasia system according to AVMA Guidelines for the Euthanasia of Animals and all procedures complied with the animal care guidelines from the First Affiliated Hospital of Zhengzhou University.

### Immunohistochemistry

Immunohistochemistry was performed as previously described [29]. Sections were incubated with antibodies: phospho-Stat3 (Ser473) (diluted 1:100; Cell Signaling Technology) and Ki-67 (diluted 1:50). Expression levels were scored using the mean optical density (AOD) by Image-Pro Plus 6.0.

### TUNEL assay

We used TUNEL staining using a TUNEL Apoptosis Assay Kit (keyGen BioTECH, Nanjing, China) following the manufacturer’s instruction.

## Results

### CircRNA profiling of NKTCL patients’ plasma

**Fig. 1:**
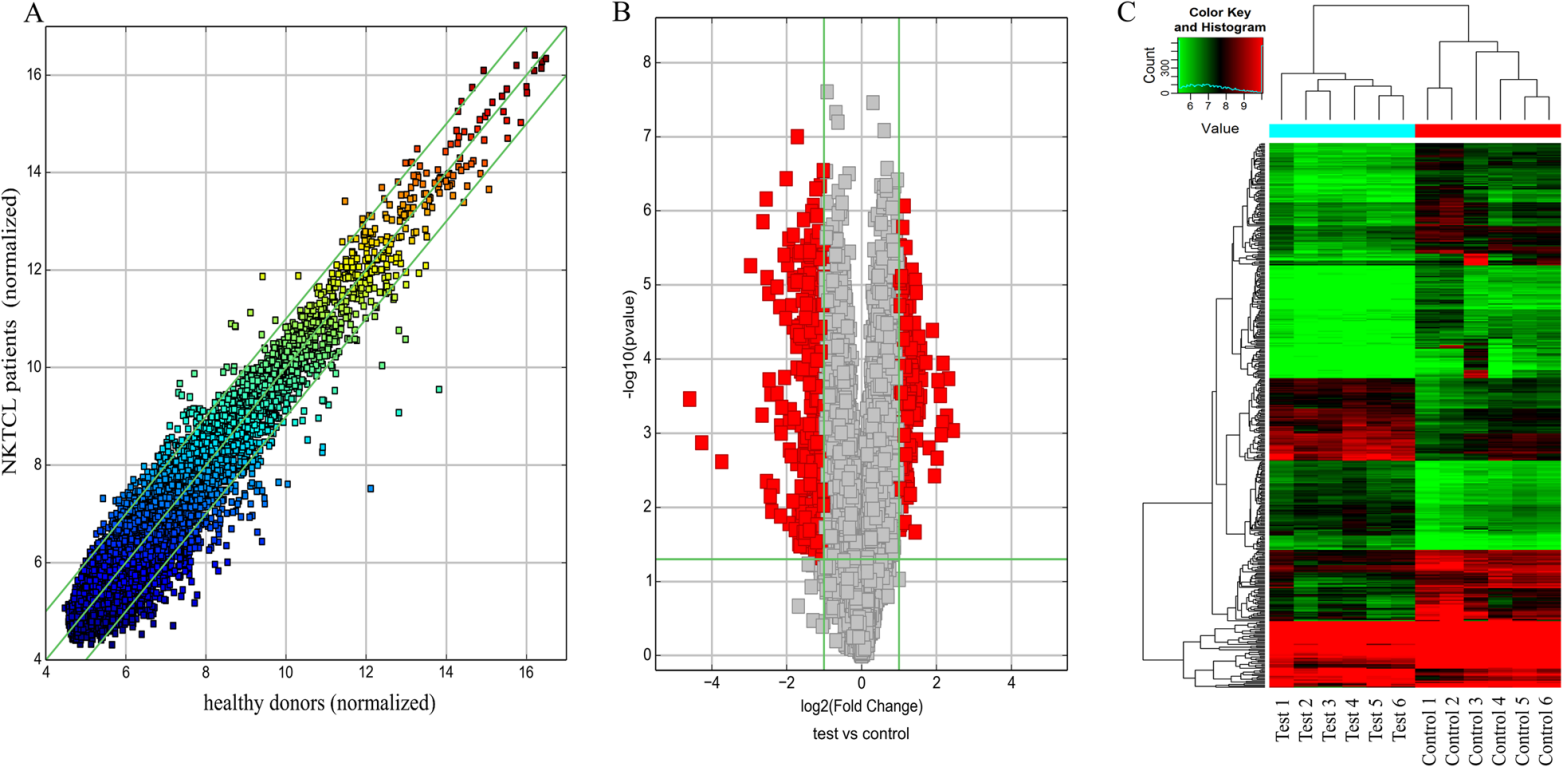
Differential expression of circRNA in NKTCL patients and healthy controls. **A** Scatter plot; The CircRNAs above the top green line and below the bottom green line indicated more than 2.0-fold change of circRNAs between the two groups. **B** Volcano plot; The vertical lines correspond to a 2.0-fold increase and decrease, respectively, and the horizontal line represents a p-value of 0.05. The red dots represent circRNAs with statistically significant differential expression. **C** Hierarchical clustering; Hierarchical clustering was performed based on all targets value. The result of hierarchical clustering shows a distinguishable circRNA expression profiling among samples

We performed microarrays to characterize the profiles of circRNAs in the plasma from 6 NKTCL patients and six healthy donors (Additional file 2). The results showed that 6137 up-regulated circRNAs and 6190 down-regulated circRNAs with differential expression (FC value > 1.0). Scatter plot, Volcano map and cluster analysis plot were drawn according to the results (see Fig. 1A–C). According to the conditions of FC value > 2.0 and P < 0.05, the differentially expressed circRNA was further screened, of which 170 were up-regulated and 188 were down-regulated. The top five upregulated circRNAs are hsa_circ_0087631, hsa_circ_0045932, hsa_circ_0052372, hsa_circ_0005037, hsa_circ_0007059, of which circADARB1 (ID hsa_circ_0005037 in circbase) was stably expressed and the difference was significant in small sample qPCR experiment (P = 0.029).

### CircADARB1 is highly expressed in the plasma of NKTCL patients

**Fig. 2:**
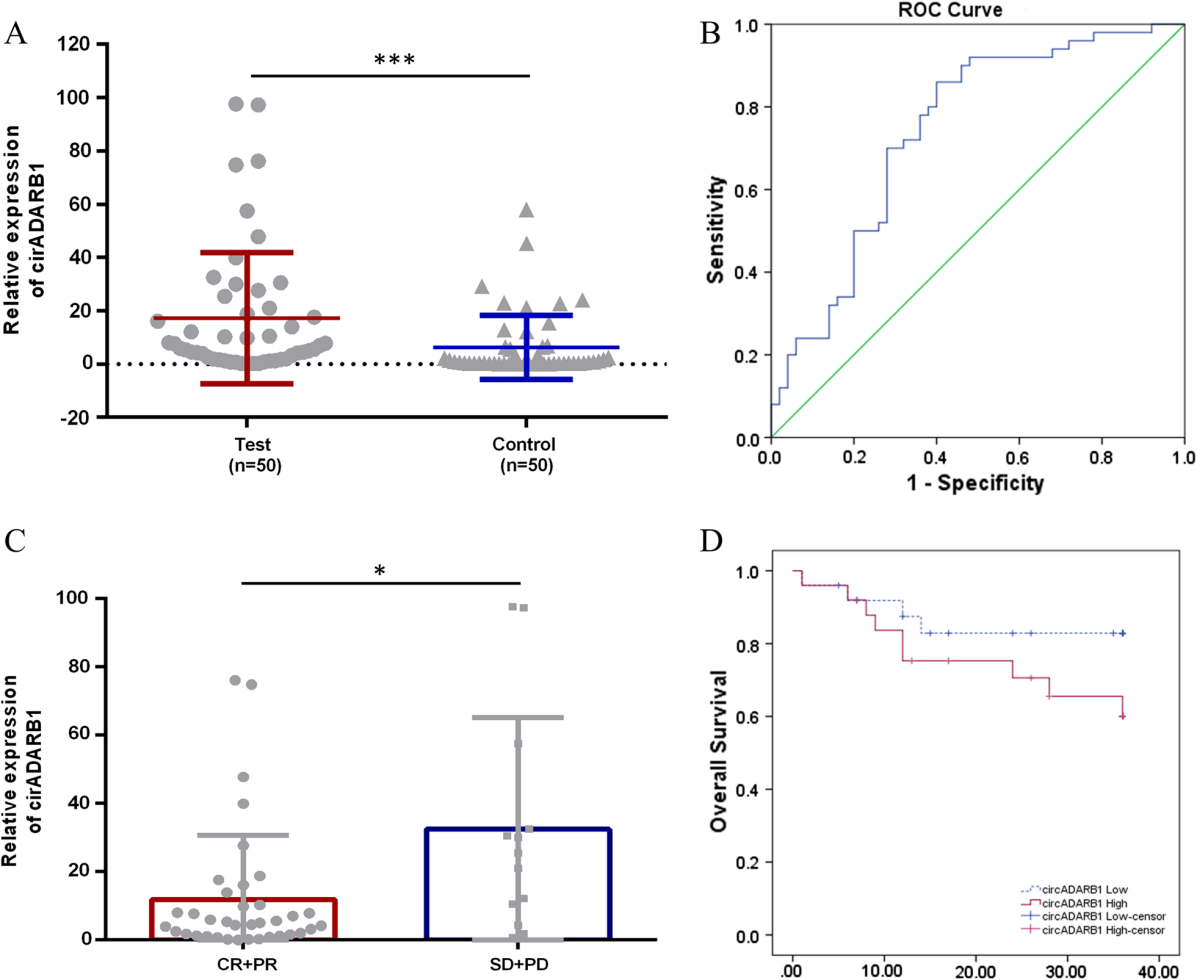
Analysis of relative expression of circADARB1 and clinical data of NKTCL patients. **A** Comparison of the relative expression of circADARB1 in the plasma of NKTCL patients and healthy controls; **B** ROC curve of circADARB1; **C** Comparison of the relative expression of circADARB1 in NKTCL patients with different efficacy; **D** three-year survival curve. *P < 0.05, ***P < 0.001

The expressions of circADARB1 in plasma of 50 NKTCL patients and 50 healthy controls were measured by qRT-PCR. The results suggested that circADARB1 was significantly up-regulated in the plasma of NKTCL patients (P < 0.001) (Fig. 2A). The receiver operating characteristic curve (ROC) was drawn, and the area under the curve (Area Under Curve, AUC) was calculated. The results showed (Fig. 2B) that the AUC was 0.742 ± 0.050 (P < 0.001), the 95% confidence interval was (0.644, 0.840), the sensitivity was 0.860, and the specificity was 0.600. The results showed that the relative expression of circADARB1 in plasma may assist the diagnosis of NKTCL.

We next evaluated the association between circADARB1 and clinical pathological parameters. The results showed that the relative expression of circADARB1 was no statistically difference grouped by age, gender, B symptoms, stage, PINK-E prognostic index and ECOG score (Table 1). But the expression of circADARB1 was associated with efficacy (classified into CR + PR and SD + PD) (P = 0.041, Fig. 2C), and the relative expression of circADARB1 was higher in patients with SD and PD. The results suggest that circADARB1 may be a potential biomarker for predicting efficacy. Using the median expression level of circADARB1 as cutoff value, 50 NKTCL patients were divided into two groups: high expression and low expression of circADARB1. Survival analysis was conducted based on follow-up data, and a 3-year survival curve was drawn (Fig. 2D). The overall survival rates of the high- and low-expression group were 60.1% and 82.9%, respectively, but the difference was not statistically significant (P = 0.153).

**Table 1:** Relationship between the relative expression of circADARB1 in plasma of NKTCL patients and the characteristics of clinical data

Clinical characteristics	Group	Cases	*P-*value
Age	< 60	43	0.584
	≥ 60	7	
Sex	Male	32	0.613
	Female	18	
B symptom	With	26	0.497
	Without	24	
Stage	I–II	38	0.525
	III–IV	12	
PINK-E	Low	40	0.933
	Intermediate and High	10	
ECOG	0–2	44	0.610
	3–5	6	
Response to therapy	CR, PR	37	0.041
	SD, PD	13	

### Knockdown of circADARB1 inhibits the proliferation and promotes apoptosis of NKTCL cell lines in vitro

**Fig. 3:**
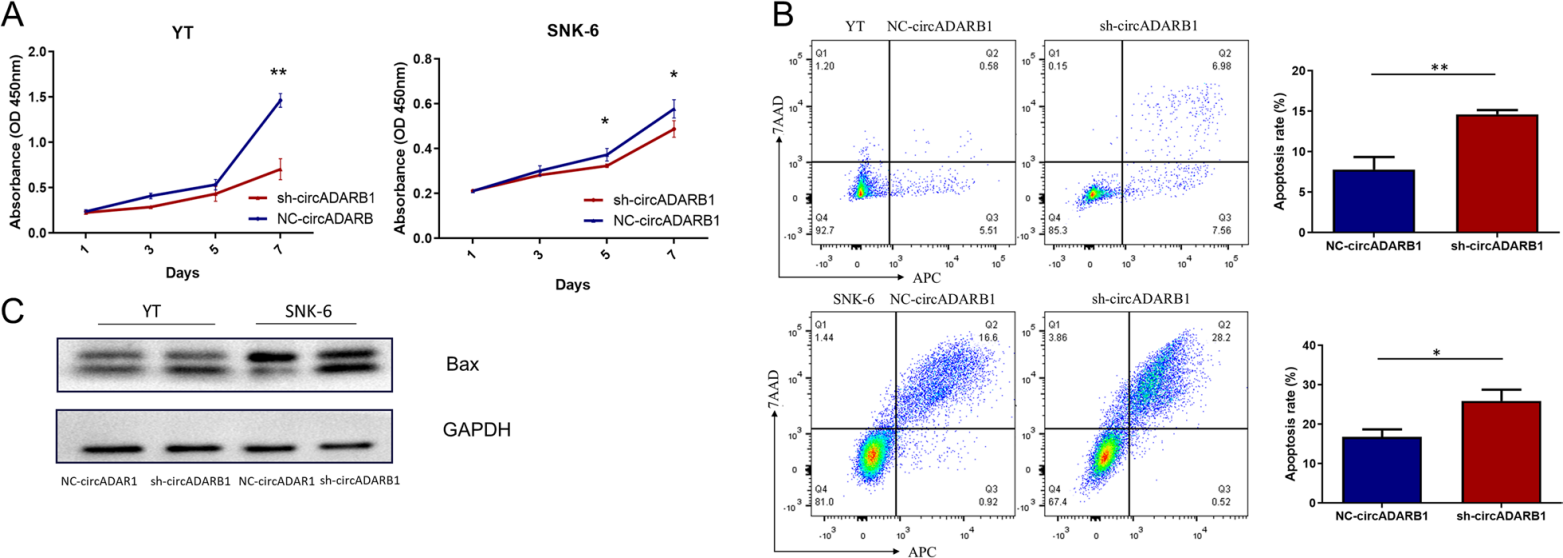
Functional experiments after transfection of NKTCL cells. **A** CCK-8 experiment in YT and SNK-6 (from 1 to 7 days) cells; **B** Apoptosis experiment results of NC-circADARB1 and sh-circADARB1 transfected YT and SNK-6 cells. **C** Western Blot detection of Bax in NC-circADARB1 and sh-circADARB1 transfected YT and SNK-6 cells. Note: sh-circADARB1 stands for the knockdown of circADARB1; NC-circADARB1 stands for transfected with control vectors. *P < 0.05, **P < 0.01

According to the results of CCK-8, it was found that the proliferation of YT cells after the knockdown of circADARB1 was significantly lower than that of the control group (Fig. 3A, P = 0.001 on day 7), and similar results were found in SNK-6 (Fig. 3A, P = 0.043 on day 5, P = 0.047 on day 7). The results showed that after knockdown of circADARB1, the proliferation of NKTCL cells decreased.

The cell apoptosis analysis showed that the apoptosis rate of YT cells [(14.63 ± 0.3033) %] with knockdown of circADRB1 was significantly higher than that of control group [(7.803 ± 0.8872) %] (Fig. 3B, P = 0.002). The results were similar in SNK-6 (Fig. 3B, P = 0.010). The western blot results showed an increase in Bax protein expression after knockdown of circADARB1 (Fig. 3C). The results indicated that knockdown of circADARB1 promoted apoptosis of NKTCL cells.

### Knockdown of circADARB1 inhibits the proliferation of NKTCL cells in vivo

**Fig. 4:**
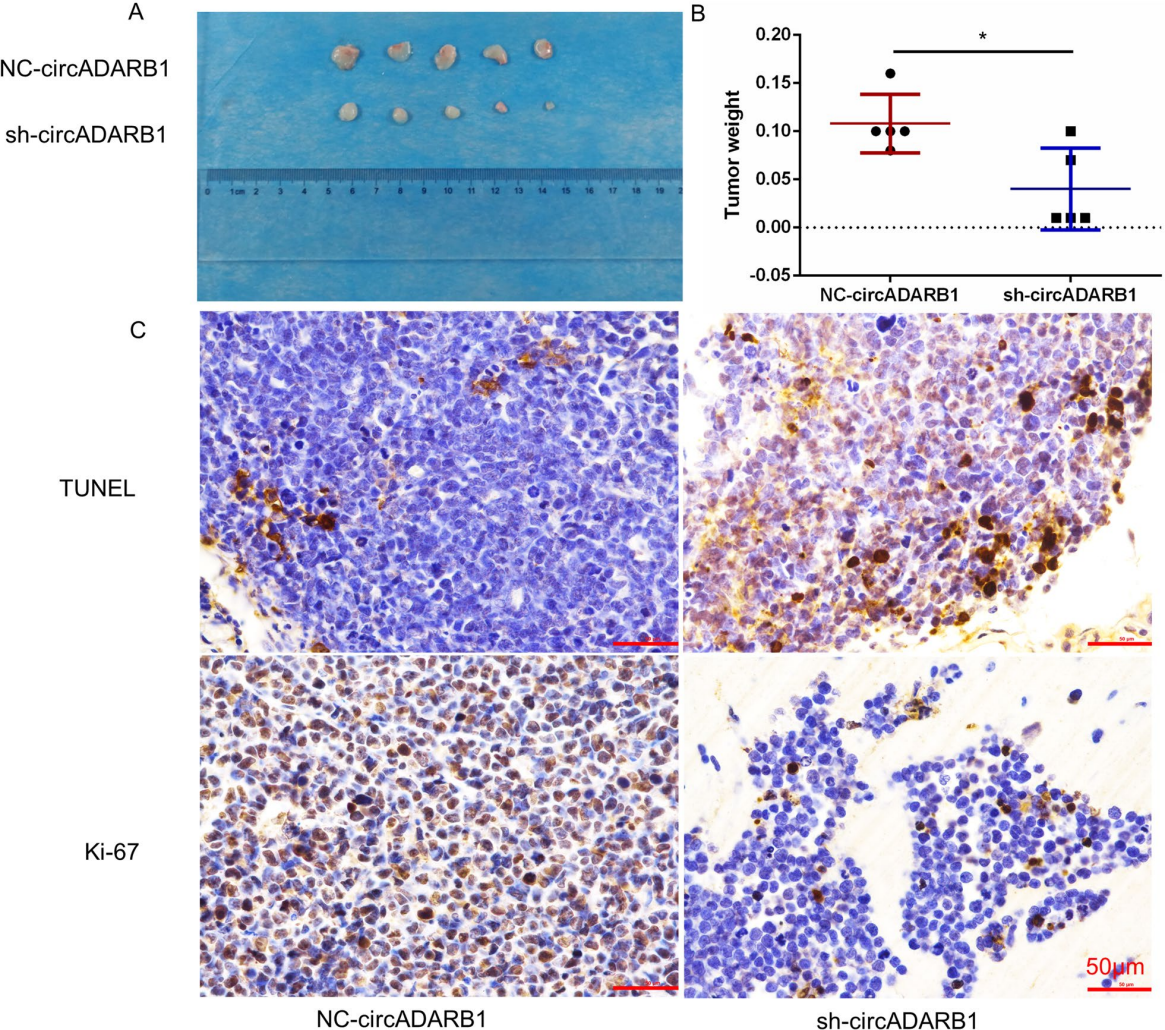
Knockdown of circADARB1 inhibits the proliferation of NKTCL cells in vivo; **A** Volume of subcutaneous tumor formation in nude mice between NC-circADARB1 and sh-circADARB1 group; **B** Comparison of the quality of subcutaneous tumor formation between NC-circADARB1 and sh-circADARB1 group; **C** Ki-67 and TUNEL assay of nude mice tumor formation experiment between NC-circADARB1 and sh-circADARB1 group. sh-circADARB1 stands for the knockdown of circADARB1; NC-circADARB1 stands for transfected with control vectors.*P < 0.05

The transfected YT cells were inoculated into the right armpit of nude mice to establish the NKTCL tumor-bearing mouse models. The tumor volumes in the knockdown of circADARB1 group were significantly smaller (Fig. 4A), and weight of tumors was lighter in comparison with control group (P = 0.019, Fig. 4B). The results show that the knockdown of circADARB1 can inhibit the proliferation of NKTCL cells in vivo. The relative expression of circADARB1 from knockdown group was significantly lower than that of the control group (P < 0.001, Additional file 1: Fig. S1A).

The results of TUNEL assay (Fig. 4C) showed that the tissues from knockdown group was positive while the control group was weakly positive (Additional file 1: Fig. S1B, P = 0.013). The Ki-67 in the knockdown group was weakly positive while the Ki-67 in the control group was positive (Fig. 4C, Additional file 1: Fig. S1C, P = 0.032).

### circADARB1 serves as a sponge for miR-214 and downstream signaling STAT3

**Fig. 5:**
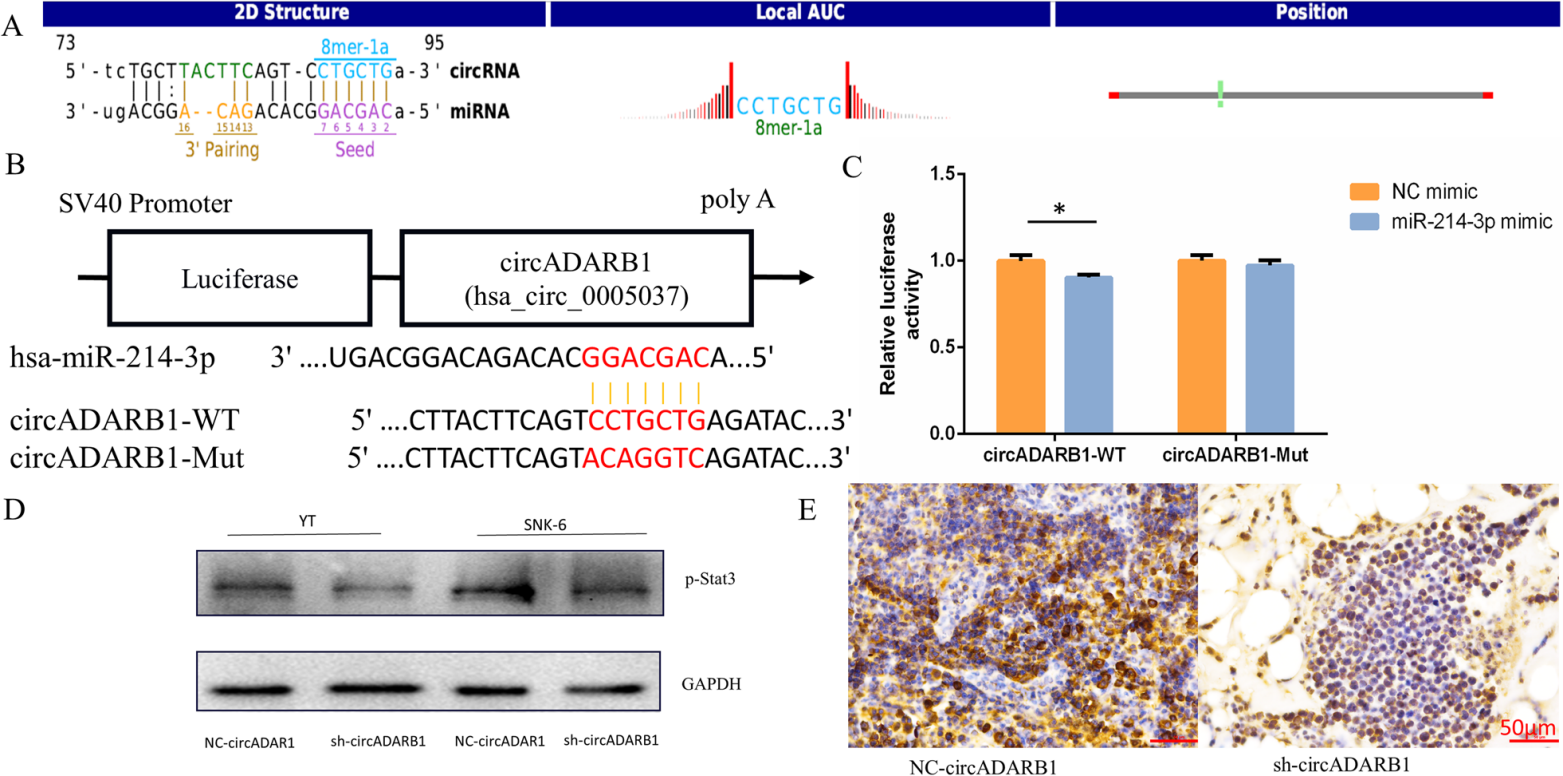
Downstream of circADARB1. **A** Analysis of circADARB1 and miR-214-3p binding sites; **B** Dual luciferase reporter design for miR-214-3p and circADARB1 target site; **C** Relative luciferase activity reported by circADARB1 and miR-214-3p dual luciferase reporter assay; **D** Western Blot detection of p-Stat3; **E** p-Stat3 immunohistochemistry in animal experiments. Note: sh-circADARB1 stands for the knockdown of circADARB1; NC-circADARB1 stands for transfected with control vectors. *P < 0.05, **P < 0.01

Through TargetScan and miRanda bioinformatics analysis, it is predicted that circADARB1 can combine with miR-214-3p (Fig. 5A) to act as a competitive endogenous RNA and play the role of “miRNA sponge”.

To further explore the relationship, wild-type and mutant plasmids of circADARB1 (Fig. 5B) were designed based on the co-binding sites of circADARB1 and miR-214-3p, and a dual luciferase reporter assay was conducted. The results showed that after co-transfection of wild-type circADARB1 and miR-214-3p mimic, the relative luciferase activity was significantly reduced (P = 0.010) comparing with co-transfection of mutant circADARB1 and miR-214-3p mimic group. There was no significant difference between the mutant and control mimic groups (Fig. 5C). The experimental results indicated that circADARB1 interacts with miR-214-3p.

We analyzed the targets of miR-214-3p using bioinformatics tools ENCORI and Targetscan and predicted that miR-214-3p could bind to STAT3 in combination with the analysis of important NKTCL signaling pathways in previous studies.

Then we detected p-Stat3 expression by Western Blot (Fig. 5D). The results showed that after knockdown of circADARB1, the expression of p-Stat3 in both YT and SNK-6 was reduced. Immunohistochemical detection of p-Stat3 was performed in nude mice in vivo model tumor tissue. It was found that p-Stat3 was strongly positive in control group and was reduced in knockdown of circADARB1 group (Fig. 5E).

## Discussion

NKTCL is a rare subtype of NHL with the characteristics of aggressive invasiveness and rapid progress. The occurrence of NKTCL has obvious geographical characteristics, and it is more common in East Asia [1]. Its incidence in China is about 11% of lymphomas [32]. NKTCL has a poor prognosis, with a median survival of 59 months and a median progression-free survival of 20 months [33]. CircRNA is a member of non-coding RNA. Due to its circular structure, it has unique biological properties that make it suitable as a biomarker. Therefore, this study aims to explore the possibility of circular RNA in NKTCL as a new molecular biomarker.

There are a lot of abnormally expressed circRNAs in the plasma or serum of tumor patients. Li et al. [34] found that many circRNAs are presented in serum exosomes and thus have the potential to be circulating biomarkers in cancer diagnosis. Hang et al. [35] found circFARSA may promote lung cancer development by sponging miR-330-5p/miR-326 and attenuating their repression of *FASN*. They also found higher abundance of circFARSA in plasma than in plasma exosomes. They therefore speculated that there may be other ways to release circFARSA into the circulation [35]. Fan et al. [36] reported that hsa_circ_0001946 is highly expressed in patients’ preoperative plasma and correlates with recurrence rates in ESCC patients. Xu et al. [37] figured out that hsa_circ_0005037 (circADARB1) is downregulated in colorectal cancer tissues. In a previous study by our group, we found that circCDYL was highly expressed in the plasma of mantle cell lymphoma patients compared to healthy donors [38].

In this study, the expression levels of circADARB1 were significantly higher in plasma of NKTCL patients and correlated with the response to therapy, i.e., the relative expression of circADARB1 was higher in plasma of patients with SD or PD. The results of our study suggest that circADARB1 may be an auxiliary diagnostic and predictive marker for efficacy.

To explore the biological function of circADARB1 in NKTCL, we performed experiments in vitro and in vivo. We found that the knockdown of circADARB1 inhibits the proliferation of NKTCL cells and promotes apoptosis. The result was similar in subcutaneous tumor xenograft in nude mice, which indicates that circADARB1 has a role in promoting tumor growth in NKTCL, and circADARB1 has the potential as a therapeutic target for NKTCL.

In this study, circADARB1 was found to bind to miR-214-3p by bioinformatic analysis, as evidenced by the use of a dual luciferase reporter assay. Previous experiments have shown that miR-214 is significantly increased in activated T cells and can reduce the expression of Pten [39]. miR-214 can bind to the 3′UTR of Twist mRNA, thereby inhibiting the expression of epithelial-mesenchymal transition related gene TWIST and regulating the metastasis of intrahepatic cholangiocarcinoma [40]. The inhibition of NF-κB increase the expression of miR-214 in hepatocellular carcinoma, suggesting that NF-κB maybe a negative regulator of miR-214 expression [41]. In different tumors, miR-214 exhibits dual effects of promotion or suppression of malignant tumor, indicating that it has a complex biological mechanism during tumorigenesis [42]. miR-214 is significantly increased in patients with Sezary Syndrome of cutaneous T-cell lymphoma [43], and miR-214 inhibits apoptosis of cutaneous T-cell lymphoma cell line HUT78 [44]. The level of miR-214 is significantly higher than that of healthy donors, and miR-214 is expected to be a therapeutic target for cutaneous T-cell lymphoma [45]. Currently, there are no reports on miR-214 in NKTCL yet.

For the downstream proteins, we predicted the target of miR-214-3p through a combination of bioinformatics and the results of important signaling pathways in the previous NKTCL research. We found the binding site between miR-214-3p and STAT3. p-Stat3 is the activated form of Stat3. In this study, knocking down circADARB1 would reduce the expression of p-Stat3. The mechanism by which p-Stat3 exerts its biological functions. Previous studies have found that in the analysis of transcriptome differences between NKTCL and normal NK cells, STAT3 is a highly differentially expressed transcription factor in NKTCL [46]. In NKTCL, p-Stat3 is highly expressed in patient samples and cell lines, indicating that p-Stat3 plays an important role in the occurrence and development of NKTCL [47]. The main reason for the abnormally high expression of p-Stat3 is the mutation of STAT3 [48] and the activation of JAK/STAT3 pathway [49]. The mutation rate of the SH2 domain of STAT3 in NKTCL was 5.9%, and cell lines with this mutation were found to up-regulate p-Stat3 [48]. Another study obtained results with higher mutation rates. The rate of missense mutations of STAT3 in NKTCL was 18.9%, and the silent mutations was 5.4%. Among all patients with STAT3 missense mutations, the expression of p-Stat3 shows higher [49]. The expression profile of JAK/STAT3 pathway in NKTCL patient tissues and normal NK cells was significantly different, and further affected multiple cell functions [50]. These studies have shown that the abnormally high expression of p-Stat3 plays an important role in the development of NKTCL. In peripheral T-cell lymphoma cell line Hut78, knocking down STAT3 reduced Bcl-XL expression and promoting apoptosis [51]. In ALK + anaplastic large cell lymphoma, a strong correlation was found between the positive expression of p-Stat3 and the expression of Survivin [52]. STAT3, as an oncogene of transcription factors, can regulate the expression of MYC, VEGFA, BCL2L1, BIRC5, HGF, MMP2, MMP9, and CDK5, suggesting its pathogenesis in NKTCL [50]. In this study, the results showed that circADARB1 regulated miR-214-3p and p-Stat3, thereby promoting the proliferation of NKTCL cells. It supplemented and expanded the previous researches on p-Stat3 in NKTCL.

This study is an attempt to find biomarkers in plasma circular RNA. It is proposed that circADARB1 may become a potential biomarker of NKTCL, which is a supplement to previous studies on NKTCL. However, this study still has certain limitations. This trial lacks the design of prospective clinical studies to verify the validity of circRNA as a biomarker. Since this study only involved Chinese NKTCL patients, there might be a population bias. In addition, the other targets or the biological mechanism of miR-214 is also not fully understood. Other biological functions of circADARB1 besides the function of “miRNA sponge” also need to be further explored.

In conclusion, circADARB1 is highly expressed in the plasma of NKTCL patients and is a potential biomarker for adjuvant diagnosis and prediction of efficacy. Knockdown of circADARB1 inhibits NKTCL cell proliferation in vitro and in vivo. circADARB1 binds up to miR-214-3p and regulates p-Stat3.

## Supplementary Information

**Additional file 1: Figure S1.** Knocking down circADARB1 inhibits the proliferation of NKTCL in vivo; (A) Relative expression of circADARB1 in nude mouse tumor tissue. (B) Average optical density (AOD) of TUNEL assay between NC-circADARB1 and sh-circADARB1 group. (C) AOD of Ki-67 between NC-circADARB1 and sh-circADARB1 group. * P < 0.05, *** P < 0.001.

**Additional file 2.** Raw data for circRNA profiling.

## Data Availability

We have attached the original data (Additional file 2).

## References

[1] Shankland KR, Armitage JO, Hancock BW (2012). Non-Hodgkin lymphoma. Lancet.

[2] Yamaguchi M, Suzuki R, Oguchi M (2018). Advances in the treatment of extranodal NK/T-cell lymphoma, nasal type. Blood.

[3] Matutes E (2018). The 2017 WHO update on mature T- and natural killer (NK) cell neoplasms. Int J Lab Hematol.

[4] Kumai T, Kobayashi H, Harabuchi Y (2016). Novel targets for natural killer/T-cell lymphoma immunotherapy. Immunotherapy.

[5] Mei M, Wang Y, Zhang M (2019). Causes of mortality in cases with extra nodal natural killer/T-cell lymphoma, nasal type: a cohort study. PLoS ONE.

[6] Somasundaram N, Lim JQ, Ong CK, Lim ST (2019). Pathogenesis and biomarkers of natural killer T cell lymphoma (NKTL). J Hematol Oncol.

[7] Kim SJ, Yoon DH, Jaccard A (2016). A prognostic index for natural killer cell lymphoma after non-anthracycline-based treatment: a multicentre, retrospective analysis. Lancet Oncol.

[8] Liu PL, Cheng ZX, Lu MP, Zhang LQ, Chen HB (2020). Misdiagnosis analysis: 120 patients with nasal extranodal NK/T cell lymphoma in head and neck. Lin Chung Er Bi Yan Hou Tou Jing Wai Ke Za Zhi.

[9] Haverkos BM, Pan Z, Gru AA (2016). Extranodal NK/T cell lymphoma, nasal type (ENKTL-NT): an update on epidemiology, clinical presentation, and natural history in North American and European cases. Curr Hematol Malig Rep.

[10] Wang L, Wang H, Li PF (2015). CD38 expression predicts poor prognosis and might be a potential therapy target in extranodal NK/T cell lymphoma, nasal type. Ann Hematol.

[11] Huang H, Zhu J, Yao M (2021). Daratumumab monotherapy for patients with relapsed or refractory natural killer/T-cell lymphoma, nasal type: an open-label, single-arm, multicenter, phase 2 study. J Hematol Oncol.

[12] Lasda E, Parker R (2014). Circular RNAs: diversity of form and function. RNA.

[13] Wilusz JE, Sharp PA (2013). Molecular biology. A circuitous route to noncoding RNA. Science.

[14] Memczak S, Jens M, Elefsinioti A (2013). Circular RNAs are a large class of animal RNAs with regulatory potency. Nature.

[15] Kolakofsky D (1976). Isolation and characterization of Sendai virus DI-RNAs. Cell.

[16] Zhang Y, Zhang XO, Chen T (2013). Circular intronic long noncoding RNAs. Mol Cell.

[17] Salzman J, Gawad C, Wang PL, Lacayo N, Brown PO (2012). Circular RNAs are the predominant transcript isoform from hundreds of human genes in diverse cell types. PLoS ONE.

[18] Ashwal-Fluss R, Meyer M, Pamudurti NR (2014). circRNA biogenesis competes with pre-mRNA splicing. Mol Cell.

[19] Chen LL, Yang L (2015). Regulation of circRNA biogenesis. RNA Biol.

[20] Rybak-Wolf A, Stottmeister C, Glazar P (2015). Circular RNAs in the mammalian brain are highly abundant, conserved, and dynamically expressed. Mol Cell.

[21] Haque S, Ames RM, Moore K (2020). circRNAs expressed in human peripheral blood are associated with human aging phenotypes, cellular senescence and mouse lifespan. GeroScience.

[22] Pan B, Qin J, Liu X (2019). Identification of serum exosomal hsa-circ-0004771 as a novel diagnostic biomarker of colorectal cancer. Front Genet.

[23] Ghods FJ (2018). Circular RNA in saliva. Adv Exp Med Biol.

[24] Du WW, Yang W, Liu E, Yang Z, Dhaliwal P, Yang BB (2016). Foxo3 circular RNA retards cell cycle progression via forming ternary complexes with p21 and CDK2. Nucleic Acids Res.

[25] Hu Y, Zhao Y, Shi C (2019). A circular RNA from inhibits the proliferation of diffuse large B-cell lymphoma by inactivating Wnt/β-catenin signaling via interacting with TET1 and miR-888. Aging.

[26] Dahl M, Daugaard I, Andersen MS (2018). Enzyme-free digital counting of endogenous circular RNA molecules in B-cell malignancies. Lab Invest.

[27] Deng L, Liu G, Zheng C, Zhang L, Kang Y, Yang F (2019). Circ-LAMP1 promotes T-cell lymphoblastic lymphoma progression via acting as a ceRNA for miR-615-5p to regulate DDR2 expression. Gene.

[28] Hong H, Li Y, Lim ST (2020). A proposal for a new staging system for extranodal natural killer T-cell lymphoma: a multicenter study from China and Asia Lymphoma Study Group. Leukemia.

[29] Agarwal V, Bell GW, Nam J-W, Bartel DP (2015). Predicting effective microRNA target sites in mammalian mRNAs. Elife.

[30] Li J-H, Liu S, Zhou H, Qu L-H, Yang J-H (2014). starBase v2.0: decoding miRNA-ceRNA, miRNA-ncRNA and protein-RNA interaction networks from large-scale CLIP-Seq data. Nucleic Acids Res.

[31] Enright AJ, John B, Gaul U, Tuschl T, Sander C, Marks DS (2003). MicroRNA targets in *Drosophila*. Genome Biol.

[32] Sun J, Yang Q, Lu Z (2012). Distribution of lymphoid neoplasms in China: analysis of 4,638 cases according to the World Health Organization classification. Am J Clin Pathol.

[33] Fox CP, Civallero M, Ko Y-H (2020). Survival outcomes of patients with extranodal natural-killer T-cell lymphoma: a prospective cohort study from the international T-cell Project. Lancet Haematol.

[34] Li Y, Zheng Q, Bao C (2015). Circular RNA is enriched and stable in exosomes: a promising biomarker for cancer diagnosis. Cell Res.

[35] Hang D, Zhou J, Qin N (2018). A novel plasma circular RNA circFARSA is a potential biomarker for non-small cell lung cancer. Cancer Med.

[36] Fan L, Cao Q, Liu J, Zhang J, Li B (2019). Circular RNA profiling and its potential for esophageal squamous cell cancer diagnosis and prognosis. Mol Cancer.

[37] Xu D, Wu Y, Wang X (2020). Identification of functional circRNA/miRNA/mRNA regulatory network for exploring prospective therapy strategy of colorectal cancer. J Cell Biochem.

[38] Mei M, Wang Y, Wang Q, Liu Y, Song W, Zhang M (2019). CircCDYL serves as a new biomarker in mantle cell lymphoma and promotes cell proliferation. Cancer Manag Res.

[39] Jindra PT, Bagley J, Godwin JG, Iacomini J (2010). Costimulation-dependent expression of microRNA-214 increases the ability of T cells to proliferate by targeting Pten. J Immunol.

[40] Li B, Han Q, Zhu Y, Yu Y, Wang J, Jiang X (2012). Down-regulation of miR-214 contributes to intrahepatic cholangiocarcinoma metastasis by targeting Twist. FEBS J.

[41] Duan Q, Wang X, Gong W (2012). ER stress negatively modulates the expression of the miR-199a/214 cluster to regulates tumor survival and progression in human hepatocellular cancer. PLoS ONE.

[42] Sharma T, Hamilton R, Mandal CC (2015). miR-214: a potential biomarker and therapeutic for different cancers. Future Oncol.

[43] Ballabio E, Mitchell T, van Kester MS (2010). MicroRNA expression in Sezary syndrome: identification, function, and diagnostic potential. Blood.

[44] Narducci MG, Arcelli D, Picchio MC (2011). MicroRNA profiling reveals that miR-21, miR486 and miR-214 are upregulated and involved in cell survival in Sézary syndrome. Cell Death Dis.

[45] Kohnken R, McNeil B, Wen J (2019). Preclinical targeting of MicroRNA-214 in cutaneous T-cell lymphoma. J Investig Dermatol.

[46] Ng S-B, Selvarajan V, Huang G (2011). Activated oncogenic pathways and therapeutic targets in extranodal nasal-type NK/T cell lymphoma revealed by gene expression profiling. J Pathol.

[47] Coppo P, Gouilleux-Gruart V, Huang Y (2009). STAT3 transcription factor is constitutively activated and is oncogenic in nasal-type NK/T-cell lymphoma. Leukemia.

[48] Küçük C, Jiang B, Hu X (2015). Activating mutations of STAT5B and STAT3 in lymphomas derived from γδ-T or NK cells. Nat Commun.

[49] Liu J, Liang L, Li D (2019). JAK3/STAT3 oncogenic pathway and PRDM1 expression stratify clinicopathologic features of extranodal NK/T-cell lymphoma, nasal type. Oncol Rep.

[50] Huang Y, de Reynies A, de Leval L (2010). Gene expression profiling identifies emerging oncogenic pathways operating in extranodal NK/T-cell lymphoma, nasal type. Blood.

[51] Verma NK, Davies AM, Long A, Kelleher D, Volkov Y (2010). STAT3 knockdown by siRNA induces apoptosis in human cutaneous T-cell lymphoma line Hut78 via downregulation of Bcl-xL. Cell Mol Biol Lett.

[52] Nasr MR, Laver JH, Chang M, Hutchison RE (2007). Expression of anaplastic lymphoma kinase, tyrosine-phosphorylated STAT3, and associated factors in pediatric anaplastic large cell lymphoma: a report from the children’s oncology group. Am J Clin Pathol.

