# Supplementary figures and images for: CircADARB1 serves as a new biomarker in natural killer T-cell lymphoma and a potential regulator of p-Stat3
Source: Cancer Cell Int. 2021 Nov 4;21:594. doi: 10.1186/s12935-021-02296-x (PMC8567645; doi:10.1186/s12935-021-02296-x)

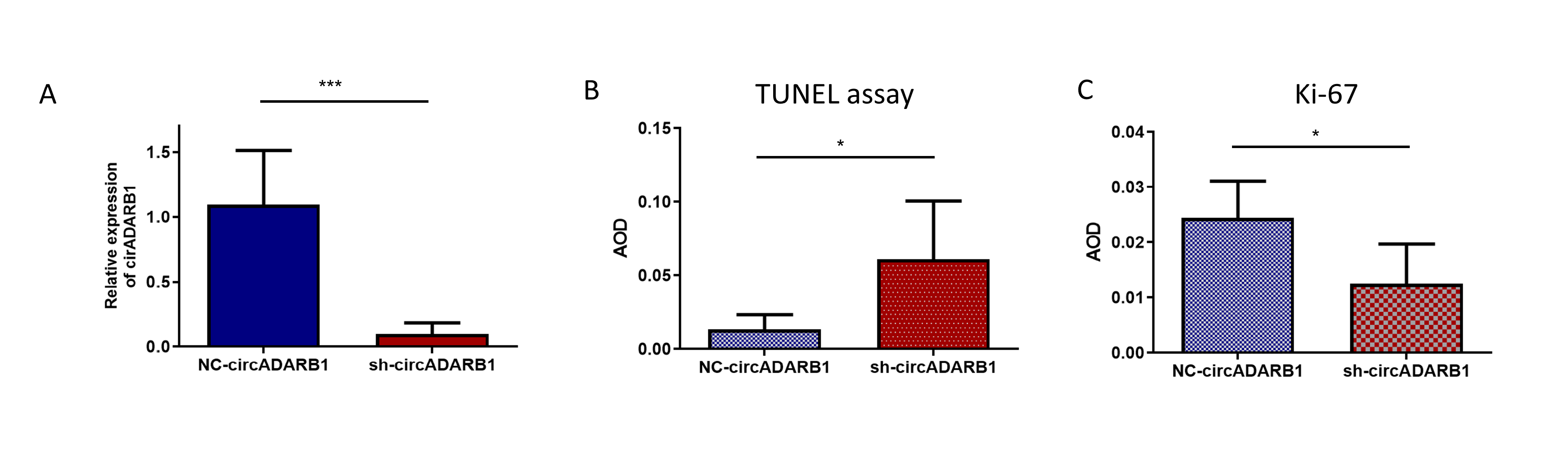

Supplement: Supplementary file 1 — Additional file 1: Figure S1. Knocking down circADARB1 inhibits the proliferation of NKTCL in vivo; (A) Relative expression of circADARB1 in nude mouse tumor tissue. (B) Average optical density (AOD) of TUNEL assay between NC-circADARB1 and sh-circADARB1 group. (C) AOD of Ki-67 between NC-circADARB1 and sh-circADARB1 group. * P < 0.05, *** P < 0.001. [file 12935_2021_2296_MOESM1_ESM.tif]
